# How do phenology, plasticity, and evolution determine the fitness consequences of climate change for montane butterflies?

**DOI:** 10.1111/eva.12618

**Published:** 2018-03-24

**Authors:** Joel G. Kingsolver, Lauren B. Buckley

**Affiliations:** ^1^ Department of Biology University of North Carolina Chapel Hill NC USA; ^2^ Department of Biology University of Washington Seattle WA USA

**Keywords:** adaptation, climate change, ectotherms, evolution, fitness consequences, phenology, plasticity

## Abstract

Species have responded to climate change via seasonal (phenological) shifts, morphological plasticity, and evolutionary adaptation, but how these responses contribute to changes and variation in population fitness are poorly understood. We assess the interactions and relative importance of these responses for fitness in a montane butterfly, *Colias eriphyle*, along an elevational gradient. Because environmental temperatures affect developmental rates of each life stage, populations along the gradients differ in phenological timing and the number of generations each year. Our focal phenotype, wing solar absorptivity of adult butterflies, exhibits local adaptation across elevation and responds plastically to developmental temperatures. We integrate climatic data for the past half‐century with microclimate, developmental, biophysical, demographic, and evolutionary models for this system to predict how phenology, plasticity, and evolution contribute to phenotypic and fitness variation along the gradient. We predict that phenological advancements incompletely compensate for climate warming, and also influence morphological plasticity. Climate change is predicted to increase mean population fitness in the first seasonal generation at high elevation, but decrease mean fitness in the summer generations at low elevation. Phenological shifts reduce the interannual variation in directional selection and morphology, but do not have consistent effects on variation in mean fitness. Morphological plasticity and its evolution can substantially increase population fitness and adaptation to climate change at low elevations, but environmental unpredictability limits adaptive plastic and evolutionary responses at high elevations. Phenological shifts also decrease the relative fitness advantages of morphological plasticity and evolution. Our results illustrate how the potential contributions of phenological and morphological plasticity and of evolution to climate change adaptation can vary along environmental gradients and how environmental variability will limit adaptive responses to climate change in montane regions.

## 1 INTRODUCTION

Organisms have responded ecologically to recent and ongoing climate changes via shifts in population size, geographic distribution and range boundaries, seasonal timing, and species interactions (Parmesan, 2006). Many of these changes are mediated by phenotypic plasticity in ecologically important traits, including development time, body size, body condition, and coloration (Chevin, Lande, & Mace, 2010; Merila & Hendry, 2014). Evolutionary responses include changes in body size, coloration, diapause cues, and thermal sensitivity (Anderson, Inouye, McKinney, Colautti, & Mitchell‐Olds, 2012; van Asch, Salis, Holleman, van Lith, & Visser, 2013; Bradshaw & Holzapfel, 2001; Higgins, MacLean, Buckley, & Kingsolver, 2014; Karell, Ahola, Karstinen, Valkama, & Brommer, 2011; Leal & Gunderson, 2012). In most cases, however, the fitness consequences of these plastic and evolutionary responses to climate change are unknown (Sgro, Terblanche, & Hoffmann, 2016; Vedder, Bouwhuis, & Sheldon, 2013).

Changes in seasonal timing (phenology) are a common response to recent climate changes in many systems (Parmesan, 2006; Thackeray et al., 2016). The fitness consequences of phenological shifts can be heterogeneous and depend on the environmental and community context (Forrest, 2016; Forrest & Miller‐Rushing, 2010; Pau et al., 2011). For example, seasonal changes associated with mean climate warming have been shown to have positive impacts on energy balance, survival, or reproduction in some systems but negative impacts in others (Anderson et al., 2012; Charmantier et al., 2008; Inouye, 2008; Ozgul et al., 2010; Tafani, Cohas, Bonenfant, Gaillard, & Allaine, 2013). The negative impacts often result from seasonal mismatches between a population and its food resources or natural enemies, and can lead to selection and evolution of traits responsive to seasonal environmental conditions (Anderson et al., 2012; van Asch et al., 2013; Boggs & Inouye, 2012; Charmantier & Gienapp, 2014).

Developmental plasticity in morphological and physiological traits is also central to organisms in seasonal environments, especially for populations that complete multiple generations each year. For example in many insects, thermal conditions for generations emerging in the early spring can be drastically different from those for generations emerging in midsummer, and plastic responses to developmental environments can lead to different phenotypes in different seasonal generations (Brakefield & Larsen, 1984; Shapiro, 1976). Developmental plasticity is central to adaptation to seasonal environments in both tropical and temperate systems (Brakefield, 1987; Tauber & Tauber, 1976; Watt, 1969). However, the extent to which developmental plasticity and evolution are adaptive and influence fitness in variable, seasonal environments is poorly understood. Will their fitness consequences be sufficient to enable populations to track sustained climate changes in the coming decades (Sgro et al., 2016)?

We explore these issues using the butterfly *C. eriphyle*, which exhibits population variation in phenology, the number of generations per year, and morphology along elevational gradients in the western United States. Detailed, mechanistic models enable us to predict the fitness implications of a key thermoregulatory trait, wing absorptivity (determined by the proportion of melanic scales), based on environmental conditions (Buckley & Kingsolver, 2012). Wing absorptivity varies among populations due to both adaptive, heritable genetic differences (Ellers & Boggs, 2004; Kingsolver, 1983b) and developmental plasticity, whereby wing absorptivity decreases with increased developmental temperatures. Our modeling in this system predicts that recent climate change has generated selection for decreased mean and increased plasticity in wing absorptivity and that the importance of plasticity varies with elevation (Kingsolver & Buckley, 2015, 2017).

Here we utilize this system to investigate how phenological plasticity interacts with morphological plasticity and evolution to determine seasonal, phenotypic, and fitness consequences of recent climate change for *C. eriphyle* populations along an elevation gradient. First, how do developmental temperatures influence phenology across the elevational gradient, and what are the consequences for morphological plasticity? Second, how does plasticity alter seasonal and elevational patterns of selection and expression of wing absorptivity? Third, we quantify the fitness consequences of phenology and morphological plasticity and evolution along the gradient, and how these enhance or limit adaptive responses to climate change. By comparing these fitness consequences to those in a best‐case scenario of “perfect” (optimal) plasticity, we also illustrate how unpredictable environmental variability limits adaptive responses to climate change and test the hypothesis that phenological and morphological plasticity will buffer fitness variation in seasonal and unpredictable environments (Merila & Hendry, 2014; Parmesan, 2006).

## 2 METHODS

**Figure 1:**
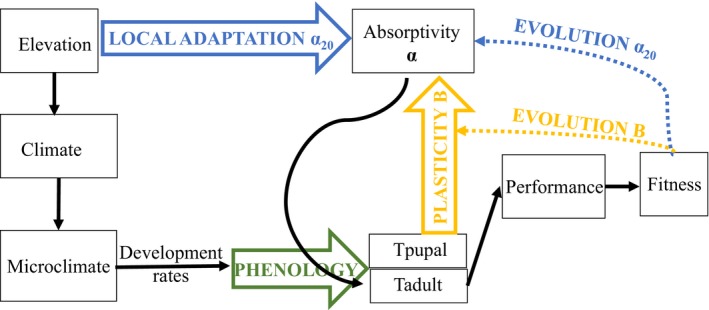
Flow diagram for the modeling framework. Climate and weather at each elevation determine the microclimatic conditions experienced by larvae, pupae, and adults at each site. Microclimate determines developmental rates of larvae and pupae, which determine phenology. The focal trait, wing melanin, is initially determined by elevation differences among sites and is also influenced by microclimate due to the plastic effects of pupal temperatures (Tpupal). We model how wing melanin influences adult temperatures (Tadult) and ultimately performance in given microclimates and then use performance to estimate fitness. Fitness differences among individuals exhibiting variation in wing melanin can generate selection and cause evolutionary changes in the mean and plasticity of wing melanin in the next generation based on Kingsolver and Buckley (2017)

We integrate microclimate, developmental, biophysical, demographic, and evolutionary models to examine the fitness implications of phenotypic and morphological plasticity (Figure 1). This modeling framework and description follows that of Kingsolver and Buckley (2017) (see Figure S1). A microclimate model is used to determine the environmental conditions experienced by larvae, pupae, and adults at three sites along an elevational gradient. Body temperatures determine developmental rates and ultimately larval, pupal, and adult phenology. The focal trait, wing absorptivity, is initially determined by elevation differences among sites (genetics) and is also influenced by microclimate due to the plastic effects of developmental (pupal) temperatures. We incorporate microclimate and thermoregulatory traits (including wing absorptivity) into a biophysical model to predict adult body temperature, behavior, and performance. We use a demographic model to relate adult performance to fitness. These fitness estimates allow us to apply a quantitative genetic model to predict phenotypic selection and evolutionary changes in wing absorptivity, and in plasticity of absorptivity, in the next generation. Below we briefly outline each component of the model (more details are provided in the SM and in (Kingsolver & Buckley, 2017)), and how the model is then used to predict phenology, plasticity, and fitness across the elevational gradient.

### 2.1 Study system

*Colias eriphyle* occurs in open habitats in the western United States at elevations from 1.4 to 3.0 km. Larvae feed on hostplants in the legume family, including alfalfa, clover, and vetch. Diapause is induced by larval photoperiod and occurs over the winter in the 3rd instar. The timing of onset of larval feeding and of the first adult flight season varies strongly with elevation (see below); and the number of adult generations per year ranges from 4‐5 at lower elevations to 2 at higher elevations (Tabashnik, 1980; Watt, Chew, Snyder, Watt, & Rothschild, 1977; Watt, Han, & Tabashnik, 1979).

We considered three sites spanning elevations of 1.8–3.0 km. Our high‐elevation site (3.0 km, 40.03N, 105.55W) is C1 of the Niwot Ridge LTER (http://niwot.colorado.edu). We examined two additional sites that are part of the National Weather Service Cooperative (COOP) Program: Cochetopa Creek (2.4 km, station 51713, 38.43N, 106.75W) and Montrose No. 2 (1.8 km, station 55722, 38.48N, 107.88W). Weather data were cleaned and filled (McGuire, Nufio, Bowers, & Guralnick, 2012). We averaged snow depth data across our study period at each site and assumed that larvae could resume development once snow melted and temperatures were permissive. We estimated snowmelt as Julian date, J = 20 at 1.8 km and J = 105 at 2.4 km (both 1961–1990 averages of data from the Western Regional Climate Center, http://www.wrcc.dri.edu/) and J = 141 at 3.0 km (averages of 1960–2010 data available from the Niwot Ridge LTER).

Rates of larval and pupal development for *C. eriphyle* depend on temperature and are well described by linear reaction norms (Higgins, 2014; MacLean, 2015). As a result, we can characterize the reaction norm for development rate *D* in terms of two parameters: the developmental zero temperature (*D*_0_) below which *D* = 0; and the accumulated degree‐days (*G*) above *D*_0_ needed to complete development. For our models, we used three different sets of values of *D*_0_ and *G* based on recent data (Higgins, 2014; MacLean, 2015): for postdiapause (4th and 5th instar) larval development (*D*_0 _= 9.22°C and *G* = 117.06°C d); for pupal development (*D*_0 _= 9.7°C and *G* = 101.9°C d); and for the entire period of (nondiapause) larval development (*D*_0 _= 11.5°C and *G* = 270.39°C d). Studies with two populations of *C. eriphyle* from different elevations yielded similar estimates, so we assumed that *D*_0_ and *G* do not change with elevation in our model.

*Colias* adults are strong flyers, and active flight is essential for courtship, mating, nectar‐feeding, oviposition, and other activities (Kingsolver, 1983a; Stanton, 1984). Flight is temperature dependent: We estimated the probability of flight as a function of operative environmental temperatures, *T*_e_: *P*_flight_ = exp(−0.5*(abs(*T*_e_ − 33.5))/5)^3.5^. The function and parameter values are based on field flight data for *C. eriphyle* in Montrose (Kingsolver, 1981). Adults behaviorally thermoregulate to achieve the body temperatures needed for flight and do not use endogenous heat production to elevate body temperatures (Watt, 1968). We assumed that butterflies select the body temperature closest to their thermal optima (33.5°C) with available body temperatures bracketed by those in full sun (lateral basking posture with wings closed and the ventral hind wing surfaces oriented perpendicular to the sun) and full shade (no direct radiation).

*Colias* adults may be exposed to short intervals of deleteriously high body temperatures (>40°C) even at high elevations, where microclimatic variation can be substantial (Kingsolver & Watt, 1983). Exposure to such high temperatures can reduce survival and fecundity: Daily heat shocks at 45°C reduce adult lifespan and egg production (Kingsolver & Watt, 1983). We modeled egg viability as an exponentially decaying function of body temperature from 1 at 40°C to 0.75 at 50°C (Buckley & Kingsolver, 2012; Kingsolver & Buckley, 2015).

Because *Colias* populations and species (including *C. eriphyle*) are adapted to local climate through differences in solar absorptivity (α) of the posterior ventral hind wings (Ellers & Boggs, 2004; Kingsolver, 1983b; Watt, 1968), our analyses here focus on variation, plasticity, and evolution of this trait. Wing solar absorptivity (α = the fraction of incident solar radiative energy that is absorbed by the wing surface) is determined by the relative proportions of pteridine (yellow or orange) and melanic (black) scales and thus spans possible values of 0.4 (all pteridine scales) to 0.7 (all melanic scales) (Kingsolver, 1983b). Two other morphological traits also influence the heat balance and body temperature of a butterfly: the length of setae on the thorax (fur thickness) and diameter of the thorax. We used a fur thickness of 0.82 mm and thorax diameter of 3.6 mm in our analyses, based on measurements for *C. eriphyle* at several sites in Colorado (Kingsolver, 1983b).

Wing melanin in *C. eriphyle* is also phenotypically plastic: Increasing temperature during pupal development decreases wing melanin (Higgins, 2014; Hoffman, 1978). The slope of the reaction norm relating wing melanin to pupal temperature is steeper (more negative) for populations at lower (1.5 km) than middle (2.1 km) elevations, and for males compared with females (J. K. Higgins, 2014; MacLean, 2015). We characterized solar absorptivity (α) for an individual by two traits: the slope of the reaction norm (*B*) relating α to the mean temperature during pupal development; and the midpoint absorptivity (α_20_), the absorptivity at a reference pupal temperature of 20°C (Kingsolver & Buckley, 2017). The mean value of α_20_ increases with elevation (Ellers & Boggs, 2002, 2004; Kingsolver, 1983b; Watt, 1968); based on data from Kingsolver (1983a), we used the initial mean starting value given by α_20 _= 0.4226 + 0.06517*E, where E = elevation in km. We estimated the mean reaction norm slope as *B* = −0.00083/°C, based on data for *C. eriphyle* males at our low elevation site (Higgins, 2014). For simplicity, we assumed that the slope does not vary with elevation. We explore both fixed and evolving values of these two traits in our simulations at each site (see below).

### 2.2 Microclimate

We estimated air temperatures (*T*_a_) at 10‐min intervals based on daily maximum and minimum temperatures using a diurnal temperature variation function incorporating sine and exponential components (Parton & Logan, 1981). Global horizontal solar radiation was calculated as a function of elevation, latitude, and longitude by discounting global extraterrestrial radiation (Campbell & Norman, 1998). Radiation was then partitioned into direct and diffuse components as a function of the atmospheric transmissivity tau [ratio of global horizontal solar radiation at surface and calculated global extraterrestrial (top of atmosphere) horizontal solar radiation] (see Figure S1). Variation in cloudiness within and among days was modeled in terms of variation in tau, using a stochastic weather generation approach (Kingsolver & Buckley, 2015).

We implemented a microclimate model (Mitchell, Beckman, Bailey, & Porter, 1975; Porter & James, 1979; Porter, Mitchell, Beckman, & DeWitt, 1973) using finite‐difference methods to solve heat balance equations describing soil temperatures at the surface and specified depths (Kingsolver, 1979; Kingsolver & Buckley, 2015). We scaled microclimate variables to plant height by estimating temperature and windspeed profiles (Campbell & Norman, 1998) using data collected at heights spanning 0.05–1.5 m in July 2012 at the subalpine site. Based on weather station data from July 2011 at this site, the mean wind speed at 0.5 m height was 0.4 m/s.

### 2.3 Developmental rates and phenological timing

**Figure 2:**
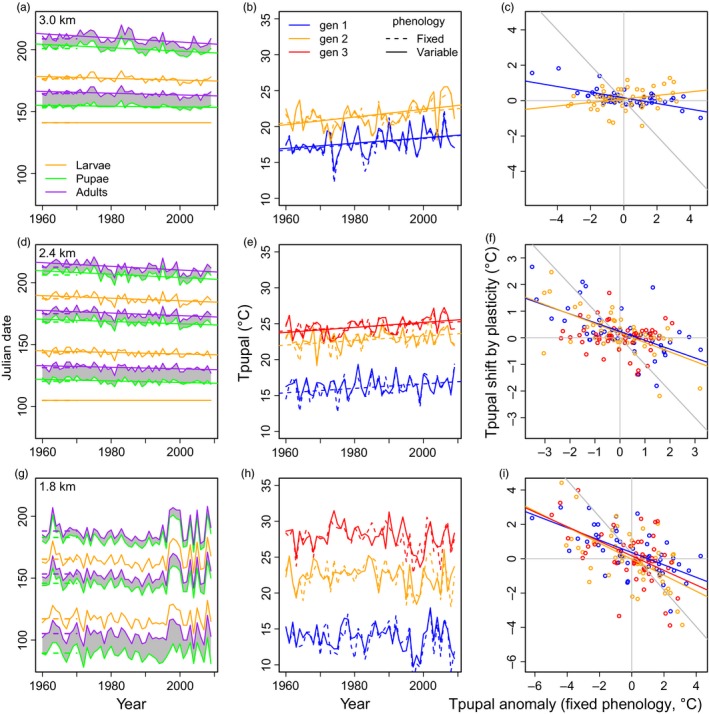
Predicted seasonal phenology and pupal temperatures across years. Climate and weather differences along the elevation gradient (top row = panels a–c: 3.0 km; middle row = d–f: 2.4 km; bottom row = g–i: 1.8 km) determine phenology and pupal temperatures (dashed lines: fixed phenology, varying phenology: solid lines). First column (panels a, d, g): the mean Julian date of appearance for larvae (orange), pupae (green), and adults (purple; gray shading: pupal duration). The short dashed line depicts the fixed phenology. Second column (panels b, e, h): The annual mean pupal temperature (Tpupal, in °C) during the first (blue), second (orange), and third (red) generations differs between the varying and fixed phenology scenarios. Third column (panels c, f, i): phenological shifts can counter increases in pupal temperatures in warm years. The *x*‐axis depicts the temperature anomaly each year during the average dates of pupation (i.e., if the average Julian dates for pupation are 150–155, average T for days 150–155 each year – average T for days 150–155 across all years). Thus, positive values indicate that temperatures during the fixed phenology are warmer than average. The *y*‐axis depicts the shift in pupal temperatures resulting from the varying phenology (i.e., Tpupal for varying phenology – Tpupal for fixed phenology). A gray line corresponds to phenology perfectly tracking pupal temperatures (slope = −1). We depict significant (*p* < .05) temporal trends

Because *Colias* larvae and pupae typically occur on the shady undersides of leaves on the hostplant, we assumed that larval and pupal temperatures were equal to air temperatures in the sun at plant height (1.8 km = 50 cm; 2.4 and 3.0 km: 20 cm). We used a single sine wave approximation (see http://www.ipm.ucdavis.edu/WEATHER/ddss_tbl.html) to calculate degree‐days (*G*) based on daily maximum and minimum temperatures at each site. For the overwintering generation, we estimated when larval development resumes (as the first date with degree‐day accumulation after snowmelt, see above) as well as the onset and completion of pupation. For subsequent generations, we assumed a duration of 7 days from adult emergence to egg laying, and 5 additional days until larvae hatch (Higgins, 2014; MacLean, 2015). Field observations indicate (and our simulations correctly predict) that two generations are completed before overwintering each year at 3.0 km, three generations at 2.4 km, and four (sometimes more) generations at 1.8 km (Tabashnik, 1980; Watt et al., 1977, 1979). For comparison, we modeled two generations each year at 3.0 km and three generations at the other two sites (see Results and Figure 2).

### 2.4 Heat balance, performance, and fitness

We used a steady‐state heat flux model for *Colias* adults that was developed and field validated by Kingsolver (1983a) to predict thoracic body temperature (operative environmental temperature, *T*_*e*_) based on thermoregulatory traits (body size, basal ventral hind wing solar absorptivity, and thoracic fur thickness), behavioral posture (basking and heat‐avoidance), and environmental conditions (Buckley & Kingsolver, 2012). The model successfully predicts patterns of *T*_*e*_, flight activity time and heat‐avoidance in the field for *C. eriphyle* and other *Colias* species along an elevational gradient in Colorado (Kingsolver, 1983b; Kingsolver & Watt, 1984). Predictions of *T*_*e*_ are updated every 10 min.

Kingsolver and Buckley (2015, 2017) used this biophysical model together with a demographic model to connect microclimate and thermoregulatory traits to fitness estimates (net reproductive rate; see Figure S1 for details). Our fitness estimates are based on 500 females per generation. We simulated a date of adult emergence for each individual using a normal distribution with a standard deviation of 2 days, truncated seven days before and after our estimated date of adult emergence for the year and generation (Tabashnik, 1980; Watt et al., 1977, 1979). We calculated daily egg production for each female as the product of available flight time (assuming 50% of available time is spent ovipositing) and the rate of oviposition (0.73 eggs/min, as estimated for Colorado *Colias* (Stanton, 1984)). We multiplied daily egg production by the average of hourly viability estimates. We estimated λ by summing over days to either a duration of 5 days, reflecting the mean adult life span in the field (Watt et al., 1977, 1979), or reaching a maximum lifetime egg production of 700 (Kingsolver, 1981) as the product of survival to maturity, daily survival, and egg production (averaged across the 500 females). In the absence of other information, we assume that juvenile (egg to adult) survival is constant across seasons and elevation in our simulations (see Figure S1). This strong assumption will clearly affect the validity of predictions about absolute mean fitness across seasons and sites, but will not alter our qualitative results about the fitness consequences of developmental plasticity and evolution (see Results).

### 2.5 Selection and evolutionary response

Our microclimate, biophysical, and demographic models allow us to predict the fitness λ of an individual *Colias* as a function of climate variables and our focal trait solar absorptivity (α). For a given set of weather conditions at a site and time period, the relationship between absorptivity α and fitness λ (the fitness surface) is typically quadratic (Kingsolver & Buckley, 2015), and fitness is greatest at some intermediate, “optimal” value α_opt_. At lower values of α, butterflies with values of α below α_opt_ are less able to achieve body temperatures needed for flight and have less flight and oviposition time, lower egg production and reduced fitness; butterflies with values of α above α_opt_ experience deleteriously high body temperature more frequently, and have greater mortality, less flight time and egg produce, and hence reduced fitness. Differences in weather conditions among sites and time periods change the value of α_opt_ and curvature of the fitness surface quadratic (Kingsolver & Buckley, 2015). Variation in absorptivity in a population causes variation in fitness, resulting in phenotypic selection (Kingsolver & Buckley, 2017).

We used a simple quantitative genetic model to predict selection and evolution of the two traits that determine the solar absorptivity of a butterfly—α_20_ and *B*—in response to climate (Kingsolver & Buckley, 2017). We used estimates of the phenotypic standard deviation of α for *C. eriphyle* (0.062) reported by Kingsolver (1983a) for population samples taken in 1980. Ellers and Boggs (2002) used parent–offspring breeding experiments to estimate the narrow‐sense heritability *h* ^2^ of wing melanin for *C. eriphyle*, yielding *h* ^2^ = 0.43 for males and 0.36 for females (Ellers & Boggs, 2002); we used a *h* ^2^ value of 0.40 for α_20_ in our simulations. We used data for full‐sib families of *C. eriphyle* from a midelevation population (2.3 km) to estimate the phenotypic standard deviation of *B* as 0.083 (Higgins, 2014). In the absence of information about heritability of *B* or about the phenotypic or genetic covariance between α_20_ and *B*, we assumed that *h* ^2^ = 0.4 for *B* and that α_20_ and *B* are uncorrelated. We also assumed that selection is sufficiently weak so that the heritabilities and phenotypic and genetic variances do not change with time (Lynch & Lande, 1993). Finally, we assumed no gene flow among populations.

Our models predict the fitness function relating solar absorptivity α to fitness for a given site and year. Combined with estimates of the phenotypic mean and variance, we estimated the (unstandardized) directional selection gradients β for both α_20_ and *B*, and used the heritability *h* ^2^ to predict the evolutionary responses to selection in the next generation in these traits (Lande & Arnold, 1983). Note that the evolutionary response to selection is directly proportional to *h* ^2^ in this model (e.g., letting *h* ^2 ^= 0 for trait *B* results in no evolution of plasticity); the simulations we describe in the next section explore how this and other scenarios influence our model predictions (see below).

### 2.6 Model predictions

We used our model simulations to explore three main issues for the three sites along the elevational (climatic) gradient over the past 50 years (1960–2010). First, we considered how developmental temperatures influence seasonal phenology and the temperatures experienced by pupae and adults in each generation. In particular, we compared the effects of varying phenology to those of fixed phenology, in which the timing of each life stage and generation occurs on the same calendar date each year (fixed at the mean predicted date for each stage and generation at the site across years). Second, we explored the effects of developmental plasticity on seasonal patterns of solar absorptivity (wing melanin), and its consequences for variation in selection and mean population fitness. We used these to identify the optimal value of absorptivity (α_opt_) and the mean pupal temperature for each generation within a year for each site. The relationship between α_opt_ and mean pupal temperature for a given year represents the optimal reaction norm for that year, which we call “perfect plasticity.” We assessed how optimal reaction norms vary among years and sites, and how these compare to the evolving reaction norms predicted by our evolutionary simulations.

Third, we explored different scenarios for phenology, plasticity, and evolution along the gradient, and how these influence the geometric mean population fitness (across generations) for each year. We considered six scenarios:

1. Constant absorptivity: α_20_ remains constant at its initial value, and there is neither plasticity nor evolution.
2. Observed plasticity: Plasticity occurs at its initial value, but neither α_20_ nor *B* evolves.
3. Evolution of absorptivity: α_20_ evolves, but there is neither plasticity nor evolution of plasticity.
4. Evolution with observed plasticity: α_20_ evolves, and plasticity is fixed at its initial value.
5. Evolution of plasticity: Both α_20_ and *B* evolve.
6. Perfect plasticity: The optimal absorptivity (α_opt_) is expressed in each generation each year.

We considered each of these scenarios for both the variable and fixed phenology cases. By comparing the results to the perfect plasticity case (which by definition will have the highest mean fitness), we can quantify how phenology, plasticity, evolution, and the evolution of plasticity contribute to adaptation to changing climates.

## 3 RESULTS

### 3.1 Effects of phenological plasticity on seasonal timing and temperatures

The predicted seasonal patterns of emergence vary across life stages, generations, and the three sites along the elevational gradient (Figure 2a,d,g). At the high‐elevation site, postdiapause larval and pupal development is delayed by the date of snowmelt and only two generations each year are possible. At middle and lower sites, postdiapause development and adult emergence occur much earlier, and three (or more) generations can occur each year. The duration of larval and pupal development (Figure 2, gray shading) decreases as temperatures warm through the season. The predicted date of adult emergence has advanced significantly (*p* < .05, linear regression) since 1960 for each generation at the 2.4 km and 3.0 km sites. The relationship is weak due to seasonal and annual variation in temperature. This variation is especially pronounced at the low site, where there is no phenological trend.

Variation in phenology generates variation in the predicted mean pupal temperatures experienced each generation at each site (Figure 2b,e,h). Unsurprisingly, pupal temperatures are lower for the first than for subsequent generations at each site, and there is substantial annual variability in pupal temperatures at all sites. However, mean pupal temperatures in the first generation actually increase with increasing elevation due to the differences in phenology among sites. There are larger differences in pupal temperature between generations at lower than higher elevation sites.

Predicted pupal temperatures at the 3.0 km site have increased significantly (*p* < .05, linear regression) since 1960 for all generations, regardless of whether phenology varies. The advancing phenology at the 3.0 km sites has not substantially altered the ~2°C increase in pupal temperatures since 1960. Environmental tracking has been more effective at the 2.4 km site: Pupal temperatures have increased significantly (*p* < .05, linear regression) across all generations when phenology is fixed, but the increase is only significant for the third generation if phenology is variable. Pupal temperatures have not shifted over time at the thermally variable 1.8 km site.

We next consider how phenological shifts influence predicted pupal temperatures by examining interannual variability (Figure 2c,f,i). This addresses whether phenological shifts enable tracking of the thermal niche. Warm years (positive anomaly in pupal temperature at fixed dates) accelerate development and result in pupation occurring earlier. Consequently, pupal temperatures can be cooler (relative to pupal temperatures at the fixed dates) in warm years, which may result in the thermal plasticity of wing melanin being maladaptive. This phenological tracking of the thermal niche is incomplete: Slopes are all shallower than the case of perfect phenological tracking (Figure 2c,f,i). The tendency for pupal temperatures to be cool in warm years due to phenological tracking is particularly pronounced at 1.8 km sites. The negative slopes are significant and steep across all generations at 1.8 km (estimates ± *SE* by generation: 1st = −0.37 ± 0.07; 2nd = −0.46 ± 0.09; 3rd = −0.44 ± 0.10; all *p* < .001, linear regressions). Slopes decline with elevation, but remain significant for the early generations at the 2.4 km (1st = −0.33 ± 0.08; 2nd = −0.35 ± 0.07; all *p* < .001) and 3.0 km (1st = −0.15 ± 0.02; *p* < .001, linear regressions) sites. At 3.0 km, the second generation exhibits a significant contrary trend because phenological advancements in the first generation are limited by snowmelt at this site, with slightly warmer pupal temperatures in warm years. The cool pupal temperatures in warm years predicted by our simulations illustrate the importance of considering phenological shifts and how they can alter patterns of plasticity and selection on subsequent life stages.

### 3.2 Consequences for plasticity and selection

**Figure 3:**
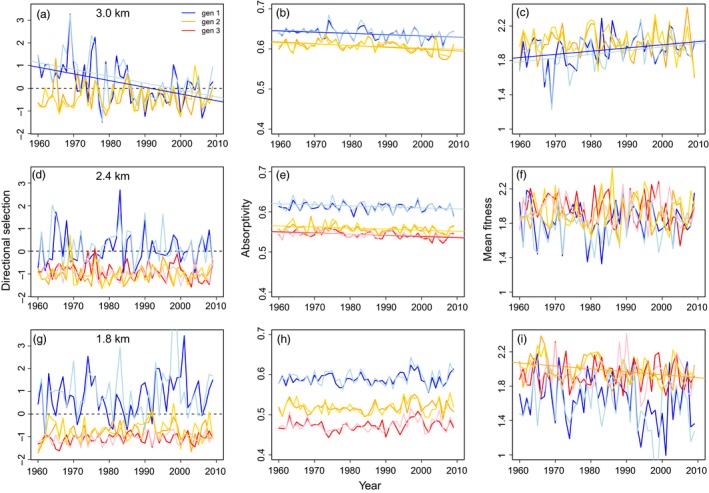
Predicted directional selection, mean absorptivity, and mean fitness across years. For the case with observed plasticity and no evolution, we depict the directional selection gradient (β) on absorptivity (wing melanin, panels a, d, g), mean absorptivity (panels b, e, h), and mean fitness (panels c, f, i) across elevations (top row = panels a–c: 3.0 km; middle row = d–f: 2.4 km; bottom row = g–i: 1.8 km). We depict each elevation under both variable (blue: first, orange: second, red: third) and fixed phenology (light blue: first, gold: second, pink: third). We depict significant (*p* < .05) temporal trends

These patterns of phenology and pupal temperature have important consequences for the predicted patterns of trait expression and selection on wing melanin (solar absorptivity; Figure 3). Here we focus on predictions for the case with observed plasticity with no evolution of either mean or plasticity in absorptivity. At the high site, mean absorptivity is high, but the difference in absorptivity between generations is modest. Absorptivity tends to decrease across years due to increases in pupal temperature (Figures 2, 3). Differences in absorptivity among generations are greater at the middle and especially the low site, reflecting greater seasonal variation in pupal temperatures. As a result, the predicted phenotypic consequences of plasticity are greater at lower than at higher elevations (Figure 3b,e,h) (Kingsolver & Buckley, 2017). The effects of varying phenology on mean absorptivity are relatively modest and are inconsistent in direction across generations and years (Figure 3b,e,h). Overall, varying phenology tends to decrease interannual variance in mean absorptivity at the low elevation site, but has no consistent effect on variance in absorptivity at the mid‐ or high‐elevation sites.

Realized seasonality across the elevation gradient also influences variation in directional selection on absorptivity across generations (Figure 3a,d,g). At all sites, cool conditions in the first generation generally select for increased absorptivity (positive directional selection), with substantial interannual variability. Selection during the first generation at the high site shifts from positive to little or negative selection over the time period 1960–2010. In contrast, selection is consistently negative in the second and third generations through this period, especially at the middle and low sites. This suggests that, despite adaptive plasticity in absorptivity, the direction of selection varies consistently between generations in response to seasonal variation, favoring the evolutionary of greater plasticity (Kingsolver & Buckley, 2017). Differences in selection resulting from varying phenology are inconsistent in direction across years (Figure 3a,d,g). Overall, varying phenology tends to decrease interannual variance in selection at the low elevation site, with little effect on variance in selection at the mid‐ or high‐elevation sites.

**Figure 4:**
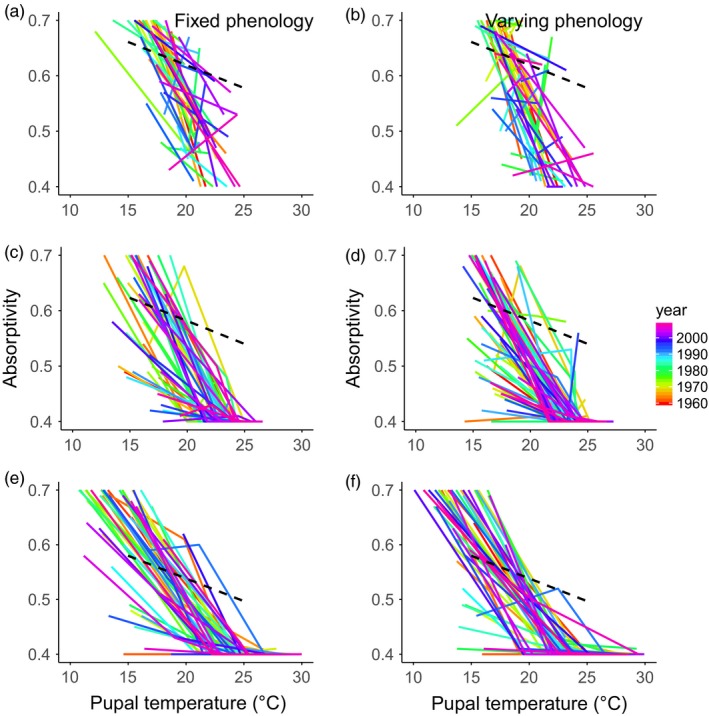
Optimal reaction norms. Optimal reaction norms (optimal absorptivity as a function of mean pupal temperature in each generation) across years for the fixed (panels a, c, e) and variable (panels b, d, f) phenology for each elevation (top row = panels a–b: 3.0 km; middle row = c–d: 2.4 km; bottom row = e–f: 1.8 km). The observed reaction norm (dashed black line) is also included

By finding the absorptivity value that yields the highest fitness in each generation, we can also predict the optimal reaction norm (optimal absorptivity as a function of mean pupal temperature) for each year at each site (Figure 4). The slopes of the optimal reaction norms are highly variable among years, especially at the higher elevation sites: Indeed in some years, the slope of the optimal reaction norm is positive rather than negative. At the low site, the slopes of the optimal reaction norms are more consistent, but there is substantial variation among years in the position (i.e., α_20_) of the reaction norms (Figure 4a,c,e). Importantly, the optimal reaction norms are consistently steeper (more negative slope) than the observed reaction norm (dashed black line), especially at the lower sites. As a result, there is consistent selection for evolutionary increases in the magnitude of developmental plasticity in this system (Kingsolver & Buckley, 2017). The effects of variable vs fixed phenology on optimal reaction norms are heterogenous, especially at the higher elevation sites (Figure 4). Varying phenology does not have consistent effects on either the slope or position of the optimal reaction norms across years at any of the sites.

### 3.3 Fitness consequences of phenology, plasticity, and evolution

The predicted changes in mean population fitness in each generation reveal several interesting patterns (Figure 3c,f,i). First, mean fitness is generally lower in the first than in subsequent generations, especially at the low site. Several predicted temporal trends in fitness are evident: Mean fitness increases over time in the first generation at the high site and decreases over time in the second generation at the low site (Figure 3c,f,i). These trends are the consequences of warmer spring conditions at higher elevations, and hotter summer conditions at low elevations (Figure 2). Second, annual variability in mean fitness is large at all sites, especially in the first generation: Annual variability is larger than the temporal trends in mean fitness. The effects of varying phenology on variance in mean fitness are relatively small in magnitude and inconsistent in direction.

**Figure 5:**
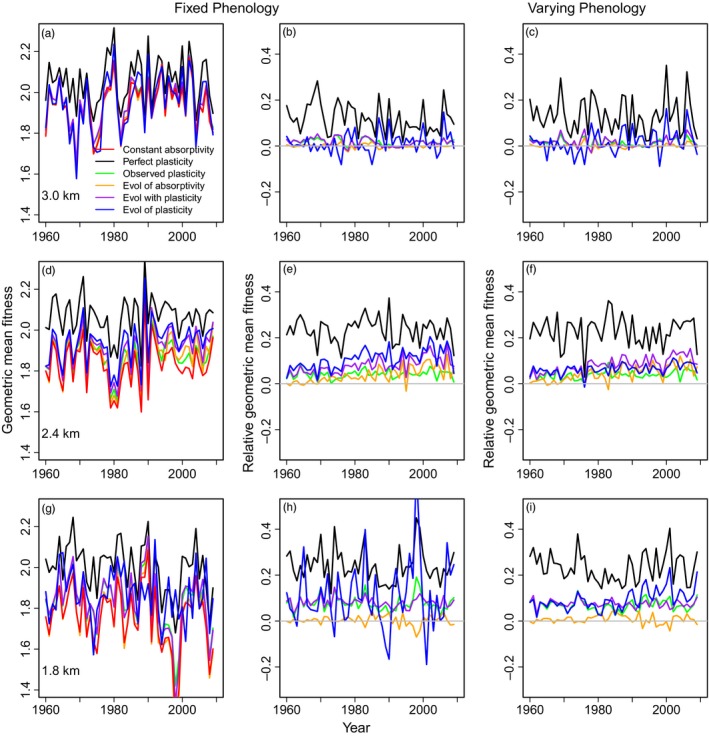
Geometric mean population fitness as a function of year. The population fitness under fixed phenology for each site (top row = panels a–c: 3.0 km; middle row = d–f: 2.4 km; bottom row = g–i: 1.8 km) varies among scenarios (see methods and legend, left column). We additionally depict geometric mean fitness relative to the case of constant absorptivity for the cases of fixed phenology (panels b, e, h) and varying phenology (panels c, f, i)

Finally, we investigate how plasticity, evolution, and phenology alter geometric mean population fitness for each year over the time period 1960–2010. Across all plasticity and evolution scenarios, fitness varies substantially across years due to seasonal and annual variation in environmental conditions (Figure 5). We consider the scenarios relative to the case of constant absorptivity (no plasticity nor evolution), which generally (but not always) confers the lowest fitness. We first consider the case of fixed phenology (Figure 5b,e,h). Across the elevation gradient, perfect plasticity increases fitness 13.3%–23.3%, with greater augmentation of fitness occurring at lower elevations. At the low elevation site, evolution of absorptivity did not increase fitness in the absence of plasticity. Observed plasticity (both including and omitting evolution of absorptivity) consistently increases fitness.

We predict that the evolution of plasticity will have variable fitness implications. At the middle elevation site, fitness tends to increase over time relative to the scenario of constant absorptivity for the scenarios that include plasticity and evolution. Evolution of absorptivity (with observed plasticity and including evolution of plasticity) tends to increase fitness over the scenarios of observed plasticity without evolution and evolution of absorptivity. In contrast at the high‐elevation site, the contributions of plasticity, evolution of absorptivity, and evolution of plasticity to increasing mean fitness are variable with predicted fitness fluctuating near zero across time.

Phenological shifts generally produce slight increases in predicted relative mean fitness across years but also increase interannual variance. Varying phenology has little influence on relative fitness at the high‐elevation site (Figure 5c,f,i). At the midelevation sites, relative fitness is similar among scenarios to the fixed phenology case, but the increase in the fitness advantage relative to constant absorptivity over time is reduced. At the low elevation site, interannual variation is reduced for the scenario of evolution of the mean and plasticity of evolution. This scenario exhibits higher relative fitness over time. These results suggest that adaptation at higher elevations is strongly limited by climatic variability and unpredictability.

## 4 DISCUSSION

### 4.1 Elevational gradients in phenology and phenotypes

Mean environmental temperatures decline with increasing elevation, and population divergence in thermally adaptive traits along elevational gradients has been widely documented. For organisms with multiple generations per year, however, patterns of climatic variation and adaptation along elevational gradients are less straightforward. Because seasonal temperatures affect developmental rates, populations at lower elevation can reach key life stages earlier, altering both seasonal phenology and the environmental conditions experienced within and across generations. Our simulations for *Colias* predict that, in the first generation each year, mean pupal temperatures are actually lower at low than at high‐elevation sites, as a result of earlier spring snowmelt and emergence at lower elevations (Figure 2). This elevational pattern is reversed in subsequent generations. One consequence is that differences in mean pupal temperatures between generations decline with increasing elevation: Thus within years, variation in pupal and adult temperatures is much greater at low than at high elevations (Kingsolver & Buckley, 2017; MacLean, Kingsolver, & Buckley, 2016).

Phenological shifts resulting from the temperature dependence of development may limit the effectiveness of plastic responses to climate change (Thackeray et al., 2016). Phenological tracking can result in early developmental stages experiencing cooler temperatures in warm years (Figure 2). This may result in cold damage (Boggs & Inouye, 2012; Inouye, 2008). In our system, cooler pupal temperatures in the first generation may lead to increased absorptivity in relatively warmer years; this maladaptive response results from the time lag between the environmental cue (pupal stage) and the selective environment (adult) (Kingsolver & Huey, 1998; Moran, 1992). More generally, developmental plasticity that evolved in more constant environments prior to accelerating climate change may be maladaptive in the variable environments associated with climate change (Chevin et al., 2010; Lynch & Lande, 1993; Merila & Hendry, 2014).

These patterns of phenology and pupal temperature have important consequences for phenotypic variation in wing melanin. As observed in the field for *C. eriphyle* (MacLean, 2015), predicted mean absorptivity declines from the first to later generations, as a result of developmental plasticity (Figure 3). However, this seasonal decline in absorptivity is much greater at lower elevations, such that the elevation cline in absorptivity is steeper in later (mid‐late summer) generations (Kingsolver & Buckley, 2017; MacLean, 2015; MacLean et al., 2016). As a result, the contribution of plasticity to seasonal variation in wing melanin declines with increasing elevation in this system.

The predicted effects of climate warming on mean absorptivity also vary with elevation in this system. For example at high and midelevations, mean absorptivity declines in both spring and summer generations over the 1960–2010 time period (Figure 3), but there is no temporal trend at the low site. These plasticity responses are the result of the increasing pupal temperatures over this time period at the high and middle (but not low) elevation sites (Figure 2). Temporal patterns in mean wing melanin in association with recent climate changes have been observed in other *Colias* species but not been evaluated in this species (MacLean, 2015; MacLean et al., 2016).

### 4.2 Elevational patterns in selection

The predicted seasonal and temporal patterns of selection on wing absorptivity also vary across the elevational gradient (Figure 3). In the first generation at the high‐elevation site, directional selection switched from positive (favoring increased absorptivity) to negative (favoring decreased absorptivity) selection over the past 50 years as a result of increasing spring temperatures during this time period (Figure 2). In contrast at low elevations, our simulations predict consistent positive selection on absorptivity in the first generation, but negative selection during the second and third generations. Despite the influence of adaptive plasticity on mean absorptivity across generations, there remains a consistent seasonal pattern of alternating selection across generations at this site. This suggests that neither phenological nor morphological plasticity is sufficient to match seasonal phenotypes to environmental conditions in this system. Our results also indicate that climate change will generate different patterns of selection and evolution on mean and developmental plasticity of wing melanin at different elevations (Kingsolver & Buckley, 2017).

Variation in directional selection on absorptivity among generations and years implies that the optimal reaction norm (the optimal absorptivity as a function of mean pupal temperature across generations for a given year and site) must vary among years (Figure 4). Interannual variation in the optimal reaction norm is particularly striking at higher elevation: The optimal reaction norm slope varies from strongly negative to strongly positive in some years. At low elevation, the optimal slope is more consistent, but there is variation in the position (optimal α_20_) of the reaction norm. These predicted patterns suggest that pupal temperature is a less accurate predictor of climatic conditions experienced by adults at higher elevations, because of greater stochastic climatic variability and a narrower range of pupal temperatures experienced at higher elevations. The lack of predictable environmental cues in this system limits the evolution of adaptive developmental plasticity at higher elevation (Hoffman, 1978; Moran, 1992).

Surprisingly, varying phenology does not reduce interannual variation in the optimal reaction norms (Figure 4). We had expected that varying phenology would generate stronger correlations between pupal and adult conditions and enhance the adaptiveness of developmental plasticity, but our results do not support this expectation. We believe that a key reason for this disconnect between pupal and adult conditions is that adult body temperatures (and flight and fitness) are much more strongly driven by solar radiative conditions and cloud cover than pupal temperatures: In the absence of high solar radiative intensities, butterflies simply cannot achieve the body temperatures needed for active flight, especially at higher elevations (Ellers & Boggs, 2004; Kingsolver, 1983b; Watt, 1968).

### 4.3 Plasticity, evolution, and fitness variation in changing environments

Our simulations predict that climate change during the past 50 years has differing effects on mean fitness for populations at different elevations: Predicted mean fitness increased in the spring generation at high elevation, but decreased in the summer generation at low elevation (Figure 3). Our high‐elevation site (3.0 km elevation) is near the upper distributional limit of *C. eriphyle* in Colorado, and populations are small and widely scattered at these elevations. As a result, recent and future climate warming may increase the upper elevational limits of this species, regardless of its phenological responses. The interannual variation in predicted mean fitness is striking at all sites, especially for the first (spring) generation, and the variation among years is substantially larger than the mean trend over this time period. The effects of variable spring weather on population abundance and fitness have been documented for many temperate and montane butterflies (Boggs & Inouye, 2012; Dennis, Kemp, & Beckwith, 1986).

A central question is whether plasticity and evolution can improve population fitness in the face of recent and future environmental change and thereby reduce the likelihood of population decline and extinction (Chevin et al., 2010; Lynch & Lande, 1993). Our simulations address this question for wing melanin in *Colias* relative to two limiting cases: a constant wing absorptivity, in which absorptivity is not developmentally plastic and does not evolve; and perfect plasticity, in which the optimal absorptivity is achieved every generation in each year (Figure 5). Even for perfectly adapted populations, there is substantial annual variation in mean fitness at all elevations as a result of seasonal and annual variation in weather. Conversely, constant wing melanin strongly reduces mean population fitness; and the evolution of wing melanin (without plasticity) causes only modest improvements in mean fitness. These results suggest that plasticity rather than evolution alone can make greater contributions to adaptive responses to climate change in this system (Charmantier et al., 2008; Sgro et al., 2016; Vedder et al., 2013).

Our simulations also predict that the adaptive effects of plasticity vary with elevation. At the low and (to a lesser extent) the midelevation sites, plasticity substantially increases mean population fitness (relative to constant wing melanin) and allowing evolution of the reaction norm further increases mean fitness as well as variance at these sites. In contrast, at the high‐elevation site, plasticity and reaction norm evolution have little effect on improving population fitness. Adaptive plasticity at higher elevations is limited by the short active season and by stochastic variation in weather at these sites (Kingsolver & Buckley, 2015, 2017). Lack of sufficient genetic variation, environmental unpredictability, and gene flow can also limit evolution of adaptive plasticity, relative to our “perfect plasticity” scenario (Levins, 1968; Moran, 1992). Further simulations in which we increase phenotypic and genetic variance in reaction norms show that even in the absence of genetic constraints, environmental unpredictability strongly limits adaptive plasticity, especially at high elevations (Kingsolver & Buckley, 2017). Collectively, our model results suggest that the predicted contributions of plasticity and evolution for population adaptation to climate change decline with increasing elevation, and that adaptation at higher elevations is strongly limited by climatic variability and unpredictability.

## CONFLICT OF INTEREST

None declared.

## DATA ARCHIVING STATEMENT

Data and R code used in this study will be available at the Dryad Digital Repository at https://datadryad.org/resource/doi:10.5061/dryad.72mr1.
