# Supplementary material for: How do phenology, plasticity, and evolution determine the fitness consequences of climate change for montane butterflies?
Source: Evol Appl. 2018 Mar 24;11(8):1231–44. doi: 10.1111/eva.12618 (PMC6099808; doi:10.1111/eva.12618)
Supplement: Supplementary file 1 [file EVA-11-1231-s001.docx]

**Supplementary material**, based on:

Kingsolver JG and Buckley LB. 2017. Evolution of plasticity and adaptive responses to climate change along climate gradients. Proceedings of the Royal Society B. DOI: 10.1098/rspb.2017.0386.


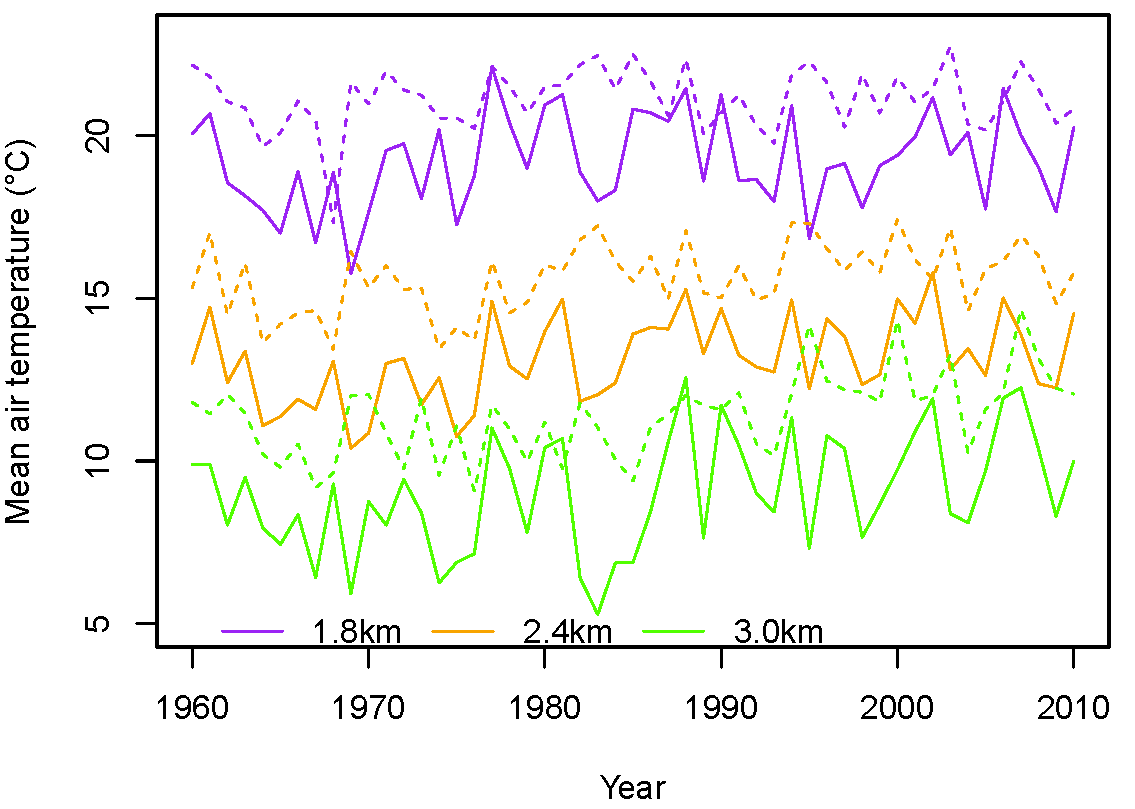


**Figure S1:** Monthly means of daily air temperatures for the focal sites between 1960 and 2010. We depict the months of June (solid) and August (dashed).


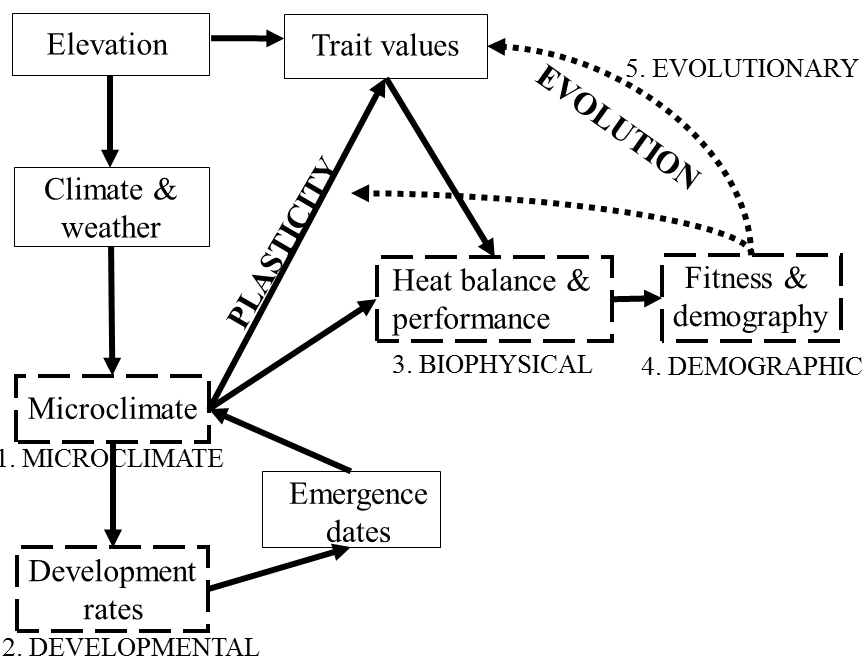


**Figure S2:** Flow diagram for the modeling framework. Boxes indicate climatic and biological metrics and model components are numbered. Elevation determines climate and weather (input: weather station data) as well as values of the focal trait, wing solar absorptivity (input: regression based on field measurements). We use (1) a microclimate model to approximate microclimatic conditions experienced by larvae, pupae and adults at each site. We then use (2) a developmental model based on lab rearing to estimate the developmental rates of larvae and pupae, which determine the seasonal timing of adult emergence. Developmental temperatures alter wing absorptivity via plastic effects (quantified by lab rearing). We use (3) a biophysical models to estimate body temperatures based on wing absorptivity and microclimate conditions. Body temperatures determine performance (flight time and egg viability based on field and lab experiments). We then use (4) a demographic model to estimate fitness based on fecundity and survival for a spectrum of values of wing absorptivity. (5) A quantitative genetic model enables estimating selection and evolutionary changes in the mean and plasticity of wing melanin in the next generation.

**Supplementary methods**

We outline each component model in figure S2 along with a justification of the model and discussion of uncertainties below.

***1. MICROCLIMATE MODEL***

Using daily maximum and minimum air temperatures (*T_a_*) from weather stations, we estimated air temperatures at ten minute intervals assuming diurnal temperature variation function incorporating sine and exponential components [1]. We scale *T_a_* across height by estimating a wind speed profile using the relationship [2,3]:


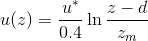
,

where *u(z)* is the wind speed (m/s) at height *z* (m), *u** is the friction velocity (m/s), *d* is the zero plane displacement (m), and the constant 0.4 is the von Karman constant. We fit this relationship to data collected at heights of 0.05, 0.25, 0.5, 0.75, 1, 1.25, 1.5m at 1 minute intervals for 12 days in July 2013 at the subalpine site (C1). We estimated *d* as the y-intercept of the relationship between *u* and ln(*z*). We then estimated surface roughness, *z_0_*, as exp(-b/n) from fitting the relationship *u*=b+n ln(*z-d*), where *u* is wind velocity (m/s) and *z* is measurement height. Using this approach we estimated of z_0_ = 0.02m at the subalpine site; we used this value in our simulations across sites. Based on weather station data from July 2013 at this site, the mean wind speed at 0.5 m height was 0.4 m s^−1^. We assumed this wind speed across sites and times because we lacked detailed wind data.

We used the following relationship to estimate air temperature and wind speed at height *z* [2,3]:


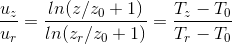
 ,

where *u_z_* and *u_r_* are the wind speeds (m/s) at height *z* (m) and reference height *r* (m), respectively. *T_z_*, *T_r_*, and *T_0_* are air and soil temperatures at height z and r and soil surface temperature, respectively.

We next estimated global horizontal solar radiation as a function of elevation, latitude, and longitude by discounting global extraterrestrial radiation as it moves through the atmosphere towards the earth surface [3]. Radiation was then partitioned into direct and diffuse components as a function of the atmospheric transmissivity *tau* [ratio of global horizontal solar radiation at surface and calculated global extraterrestrial (top of atmosphere) horizontal solar radiation]. *Tau* distributions were estimated hourly using several years of data from the NREL Solar Radiation Research Laboratory Baseline Measurement System in Golden, Colorado (1829m, 39.74N, 105.18W <http://www.nrel.gov/midc/srrl_bms/>). We used kernel density estimation to simulate a tau value for each time interval. Solar radiation was partitioned using an empirical relationship by Erb *et al*. [4], as modified for high-altitude sites in Colorado [5].

*Ta* and radiation were used as inputs for a microclimate model [2,6,7], which used finite-difference methods to solve heat balance equations describing soil temperatures at the surface and specified depths [8,9]. Modelling the soil temperature profile is necessary to accurately estimate soil surface temperatures.

***Model justification and uncertainties***

We used well-established and tested models to estimate microclimates across time and heights. However, limitations on input data introduce errors and uncertainties in our microclimate estimates. The uncertainties primarily relate to variability over time, so are unlikely to systematically introduce errors in the microclimate estimates. Errors in microclimate estimation influence estimates of developmental timing, wing absorptivity (via plasticity), and butterfly operative temperatures, but error will be minimized by basing the estimate on microclimate integrated across time.

Our use of a diurnal temperature function necessarily underestimated temporal variability at the dynamic montane sites. The function we used was parameterized for Colorado and improves estimates over simpler sine wave functions, which are commonly employed. We selected sites to possess the rare attribute of offering nearly complete daily minima and maxima air temperature data going back to 1960. Sub-daily information is not reliably available through our focal period.

Our use of mean wind speed also reduces our estimates of microclimate variability, but is unlikely to substantially influence our estimates of equilibrium butterfly operative temperatures because we use a steady-state model (see biophysical model section below). Because wind speeds can increase with elevation (depending on local topography), our assuming the mean wind speed at the subalpine site across the gradient may overestimate heat convection at the lower elevation site and overestimate heat convection at the higher site.

Radiation variation, particularly spikes, can substantially influence body temperatures at our study sites [10]. Diurnal and multiday variation in cloudiness is pronounced at the study sites and substantially influences body temperatures via altering incoming radiation. Cloudiness is extremely difficult to model, particularly at the time scales relevant to body temperatures. We thus feel that stochastically modelling atmospheric transmissivity based on empirical data provides the most realistic estimate (despite the data only being available for a relatively low elevation Colorado site).

Overall, we are confident that our microclimate estimations predict conditions experienced by butterflies more realistically than omitting microclimate temporal and height variability.

*2.* ***DEVELOPMENTAL MODEL***

We assumed that larvae overwintering as 3^rd^ instars could resume development once snow melted and temperatures were permissive. We estimated snow-melt as Julian date, J=20 at 1.8km and J=105 at 2.4km (both 1961-1990 averages of data from the Western Regional Climate Center, <http://www.wrcc.dri.edu/>) and J=141 at 3.0km (averages of 1960-2010 data available from the Niwot Ridge LTER).

We used lab data [11,12] to characterize the reaction norm for development rate D with two parameters: the developmental zero temperature (D_0_) below which D=0; and the accumulated degree-days (G) above D_0_ needed to complete development. For our models we use three different sets of values of D_0_ and G based on recent data [11,12]: for post-diapause (4^th^ and 5^th^ instar) larval development (D_0_= 9.22°C and G=117.06°C d); for pupal development (D_0_= 9.7°C and G=101.9°C d); and for the entire period of (non-diapause) larval development (D_0_= 11.5°C and G=270.39°C d). Studies with two populations of *C. eriphyle* from different elevations yielded similar estimates, so we assume that D_0_ and G do not change with elevation in our model.

Because *Colias* larvae and pupae typically occur on the shady undersides of leaves on the hostplant, we assumed that larval and pupal temperatures were equal to air temperatures in the sun at plant height (1.8km= 50cm ; 2.4 and 3.0km: 20cm). We used a single sine wave approximation (see http://www.ipm.ucdavis.edu/WEATHER/ddss_tbl.html) to calculate degree-days (G) based on daily maximum and minimum temperatures. For the overwintering generation, we estimated when larval development resumes as well as the onset and completion of pupation. For subsequent generations, we assume a duration of 7 days from adult emergence to egg laying, and 5 additional days until larvae hatch [12]. Field observations indicate (and our simulations correctly predict) that two generations are completed before overwintering each year at 3.0km, three generations at 2.4km, and four (sometimes more) generations at 1.8km [13–15]. For comparative purposes we modeled two generations each year at 3.0km and three generations at the other two sites.

***Model justification and uncertainties***

Our use of lab data to characterize the reaction norm for development rate is consistent with standard techniques. However, rearing in constant temperature conditions can underestimate how development rate responds to temperature fluctuations [16,17]. Several assumptions increase the comparability of our developmental estimates across sites and years, but reduce realism: we start the accumulation of degree days after average snowmelt date for each site and we assume constant durations between adult emergence, egg laying and larval hatching. These uncertainties are unlikely to substantially bias our estimates of developmental timing.

*3.* ***BIOPHYSICAL MODEL***

We use a biophysical model for *Colias* that was developed and field validated by Kingsolver [18] to predict thoracic body temperature (*Tb*) based on thermoregulatory traits (body size, basal ventral hind wing solar absorptivity, and thoracic fur thickness), behavioral posture (basking and heat-avoidance), and environmental conditions. We briefly describe the model, which is detailed elsewhere [18,19]. Predictions of *T_b_* are updated every 10 minutes. Adults behaviorally thermoregulate to achieve the body temperatures needed for flight, and do not use endogenous heat production to elevate body temperatures [20]. We assume that butterflies select the body temperature closest to their thermal optima (35°C) with available body temperatures bracketed by those in full sun (lateral basking posture with wings closed and the ventral hindwing surfaces oriented perpendicular to the sun) and full shade (no direct radiation). Assuming that butterflies select body temperatures bracketed by full sun and full shade eliminates the need to attempt to model microclimate availability in detail.

We describe the steady-state energy flux balance of a butterfly at rest on vegetation as


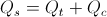


where *Q_s_* is the total solar radiative heat flux, is *Q_t_* the thermal radiative heat flux, and *Q_c_* is the convective heat flux. Conduction of heat between the body and vegetation and evaporative heat loss are considered to be negligible. The solar radiative heat flux is


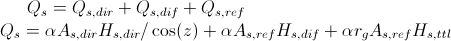


where *Q_s,dir_*, *Q_s,dif_*, *Q_s,ref_*, are the direct, diffuse, and reflected solar radiative fluxes, respectively; *H_s,dir_*, *H_s,dif_*, *H_s,ttl_*, are the direct, diffuse, and total solar radiative horizontal flux densities, respectively; *A_s,dir_*, *A_s,ref_*, *A_s,ttl_* are the direct, reflected, and total solar radiative heat transfer surface areas, respectively; *α* is wing solar absorptivity; *r_g_* is substrate solar reflectivity; and z is the zenith angle. We assume *A_s,dir_*,=*A_s,ref_*= *A_s,ttl_.*

Thermal radiative flux including both downward radiation and reflected solar radiation is estimated as follows:


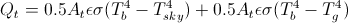


where *A_t_* is the thermal radiative heat transfer surface area, *T_b_* is the body temperature, *T_g_* is the ground surface temperature, *T_sky_* is the equivalent black body sky temperature, *ε* is butterfly thermal emissivity, and *σ* is the Stefan-Boltzman constant.

The convective heat flux is given by:


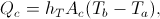


where *A_c_* is the convective heat transfer surface area, and *T_a_* is the air temperature. We assume *A_c_*=*A_t_*= *A_s,ttl_.* The total convective heat transfer coefficient, *h_T_,* is calculated as the boundary layer conductance *h_c_* and the fur layer conduction in series:


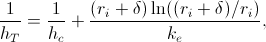


Where δ is the thoracic fur thickness and *k_e_* is the thermal conductivity of the fur. The boundary layer conductance, *h_c_*, can be estimated using the relationship between two non-dimensional numbers. The Nusselt number, *Nu*=*h_c_D/k_a_*, is the ratio of convective to conductive heat transfer, where *k_a_* is the thermal conductivity of air. We used the maximum width of the mesothorax as the characteristic dimension of the butterfly, *D*. The Reynolds number, *Re*=*uD*/*v*, is the ratio of inertial forces to viscous forces, where *u* is wind speed and *v* is kinematic viscosity. We used the *Nu*-*Re* relation for a cylinder, *Nu*=0.6*Re*^0.5^, which is a reasonable approximation for *Colias* [21]. We estimate energy flux balance in order to estimate *T_b_*.

***Model justification and uncertainties***

The biophysical model is well established and successfully predicts patterns of *T_b_ ,*flight activity time and heat-avoidance in the field for *C. meadii* and *C. eriphyle* along an elevational gradient in Colorado [18,19]. We repeated these validations at our sites using physical butterfly models (sacrificed butterflies with thermocouples embedded) and environmental sensors and dataloggers that afforded more accuracy than the initial validations [22]. There is thus relatively low uncertainty in our translation of input data into *T_b_*. Uncertainties arise due to variation in shade level available to butterflies and deviations in basking behavior from our estimates of optimal behavior. Vegetation is reasonably similar across our focal sites.

*4.* ***DEMOGRAPHIC MODEL***

We used a demographic model detailed in Buckley and Kingsolver [23] and Kingsolver and Buckley [8] along with the biophysical model (described above) to connect microclimate and thermoregulatory traits to fitness estimates (net reproductive rate). Our fitness estimates were based on 500 females per generation. We simulated a date of adult emergence for each individual using a normal distribution with a standard deviation of 2 days, truncated seven days before and after our estimated date of adult emergence for the year and generation [13–15].

We estimated annual population growth rate as a function of survival and fecundity. Mark, release, recapture studies with *C. meadii* and other *Colias* species at study sites in Colorado suggest mean adult lifespans (expected residence times) of 3-7 days that do not vary consistently with elevation (*7, 8*).

Based on these studies we assumed a daily adult survival rate, *S_daily_* of 0.6 for all populations [14,15]. We assumed that the probability of juvenile survival from egg to adult maturity, *S_mat_*, is 0.014, based on field studies of *C. alexandra*, another univoltine species with obligate winter diapause, in near Crested Butte, CO [24]. We calculated daily egg production per female, *Eggs*, as the product of available flight time and the rate of oviposition. We assumed an oviposition rate of 0.73 eggs/min and that 50% of available flight time is spent ovipositing, which was estimated for *Colias* females in Colorado [25].

We incorporated egg viability by multiplying daily egg production by the geometric mean of viability estimates across time. We modeled egg viability as an exponentially decaying function of body temperature from 1 at 40°C to 0.75 at 50°C [8,23]. We estimate λ by summing over days to either a duration of 5 days, reflecting the mean adult life span in the field [14,15], or reaching a maximum lifetime egg production of 700 [26] as


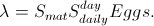


***Model justification and uncertainties***

Most of the parameters above (e.g, proportion of time spent ovipositing, rate of oviposition, daily survival and survival to maturity, adult lifespan and maximum egg production), despite being based on detailed field data, represent substantial simplifications. Although there is likely to be substantial error in our estimates of net reproductive rate, relative fitness estimates should be reasonably robust as a basis for predicting selection and evolution.

5. ***EVOLUTIONARY MODEL***

Estimates of fitness λ of *Colias* individuals as a function of wing solar absorptivity allow us to estimate (unstandardized) directional selection gradients for our two traits: α_20_, the mid-point absorptivity at a mean pupal temperature of 20°C and B, the slope of the reaction norm relating pupal temperature and wing absorptivity α. We incorporate the directional selection gradients β and heritability h^2^ in simple quantitative genetic models to predict the evolutionary responses to selection in the next generation [27]. Ellers and Boggs [28] used parent-offspring breeding experiments to estimate the narrow-sense heritability h^2^ of wing melanin for *C. eriphyle*, yielding h^2^ = 0.43 for males and 0.36 for females; we use a h^2^ value of 0.40 for α_20_ in our simulations. We used data for full-sib families of *C. eriphyle* from a middle elevation population to estimate the phenotypic standard deviation of B as 0.083 [11]. In the absence of information about phenotypic or genetic covariance between α_20_ and B, we assume that α_20_ and B are uncorrelated [29]. We also assume that selection is sufficiently weak so that the heritabilities and phenotypic and genetic variances do not change with time [30]. Finally, we assume no gene flow among populations.

***Model justification and uncertainties***

Quantitative genetic models are standard for applications such as ours where the genetic basis of the trait under selection is complex and unknown. Our estimates of h^2^ and phenotypic variance are uncertain, but additional simulations (not shown) suggest that the precise values of h^2^ or phenotypic variances have little effect on our qualitative results. Genetic correlations between the reaction norm mean and slope and gene flow could influence our projections of evolution (see Discussion in main text).

Other than evolutionary trajectories, errors propagation through our simulations should be modest. Our accounting for trait and environmental variability limits error propagation. We account for trait variability by simulating individual butterflies with traits derived from a distribution based on empirical trait means and variability. We account for variability in development and emergence time by selecting from a phenological distribution centered on mean dates. We account for environmental variability by simulating radiation from a distribution across each time period.

**References**

1. Parton WJ, Logan JA. 1981 A model for diurnal variation in soil and air temperature. *Agric. Meteorol.* **23**, 205–216.

2. Porter WP, Mitchell JW, Beckman WA, DeWitt CB. 1973 Behavioral implications of mechanistic ecology. *Oecologia* **13**, 1–54.

3. Campbell G, Norman J. 2000 *An introduction to environmental biophysics*. New York: Springer.

4. Erbs DG, Klein SA, Duffie JA. 1982 Estimation of the diffuse radiation fraction for hourly, daily and monthly-average global radiation. *Sol. Energy* **28**, 293–302.

5. Olyphant GA. 1984 Insolation topoclimates and potential ablation in alpine snow accumulation basins: Front Range, Colorado. *Water Resour. Res.* **20**, 491–498.

6. Porter WP, James FC. 1979 Behavioral implications of mechanistic ecology II: the African rainbow lizard, Agama agama. *Copeia* **1979**, 594–619.

7. Mitchell J, Beckman W, Bailey R, Porter W. 1975 Microclimate modeling of the desert. In *Heat and mass transfer in the biosphere Part I, transfer processes in the plant environment* (eds DA De Vries, NH Afgan), Washington, DC.

8. Kingsolver JG, Buckley LB. 2015 Climate variability slows evolutionary responses of Colias butterflies to recent climate change. *Proc. R. Soc. B Biol. Sci.* **282**, 20142470.

9. Kingsolver JG. 1979 Thermal and hydric aspects of environmental heterogeneity in the pitcher plant mosquito. *Ecol. Monogr.* **49**, 357–376.

10. Buckley LB, Miller EF, Kingsolver JG. 2013 Ectotherm thermal stress and specialization across altitude and latitude. *Integr. Comp. Biol.* **53**, 571–581.

11. Higgins JK. 2014 *Rapid evolution and population divergence in response to environmental change in Colias butterlies*. University of North Carolina at Chapel Hill. See http://gradworks.umi.com/36/68/3668482.html.

12. MacLean HJ, Higgins JK, Buckley LB, Kingsolver JG. 2016 Geographic divergence in upper thermal limits across insect life stages: does behavior matter? *Oecologia* **181**, 107–114.

13. Tabashnik BE. 1980 Population structure of Pierid butterflies. *Oecologia* **47**, 175–183.

14. Watt WB, Han D, Tabashnik BT. 1979 Population structure of pierid butterflies. II. A‘ native’ population of Colias philodice eriphyle in Colorado. *Oecologia* **44**, 44–52.

15. Watt WB, Chew FS, Snyder LRG, Watt AG, Rothschild DE. 1977 Population structure of pierid butterflies. *Oecologia* **27**, 1–22.

16. Kingsolver JG, Woods HA. 2016 Beyond Thermal Performance Curves: Modeling Time-Dependent Effects of Thermal Stress on Ectotherm Growth Rates. *Am. Nat.* **187**, 283–294. (doi:10.1086/684786)

17. Kingsolver JG, Higgins JK, Augustine KE. 2015 Fluctuating temperatures and ectotherm growth: distinguishing non-linear and time-dependent effects. *J. Exp. Biol.* **218**, 2218–2225.

18. Kingsolver JG. 1983 Thermoregulation and flight in Colias butterflies: elevational patterns and mechanistic limitations. *Ecology* **64**, 534–545.

19. Kingsolver JG, Watt WB. 1983 Thermoregulatory strategies in Colias butterflies - thermal-stress and the limits to adaptation in temporally varying environments. *Am. Nat.* **121**, 32–55.

20. Watt WB. 1968 Adaptive significance of pigment polymorphisms in Colias butterflies. I. Variation of melanin pigment in relation to thermoregulation. *Evolution* **22**, 437–458.

21. Kingsolver JG, Moffat RJ. 1982 Thermoregulation and the determinants of heat transfer in Colias butterflies. *Oecologia* **53**, 27–33.

22. MacLean HJ, Higgins JK, Kingsolver JG, Buckley LB. 2016 Morphological and physiological determinants of local adaptation to climate in Colias butterflies. *Conservation Physiology* **4**, 35-44.

23. Buckley LB, Kingsolver JG. 2012 The demographic impacts of shifts in climate means and extremes on alpine butterflies. *Funct. Ecol.* **26**, 969–977.

24. Hayes JL. 1980 Some Aspects of the Biology of the Developmental Stages of Colias alexandra(Pieridae). *J. Lepidopterists Soc.* **34**, 345–352.

25. Stanton ML. 1984 Short-term learning and the searching accuracy of egg-laying butterflies. *Anim. Behav.* **32**, 33–40.

26. Kingsolver JG. 1981 *Thermoregulatory strategies of Colias butterflies: adaptation in variable environments*. Dissertation. Stanford University.

27. Lande R, Arnold SJ. 1983 The measurement of selection on correlated characters. *Evolution* **37**, 1210–1226.

28. Ellers J, Boggs CL. 2002 The evolution of wing color in Colias butterflies: heritability, sex linkage, and population divergence. *Evolution* **56**, 836–840.

29. Scheiner SM, Lyman RF. 1991 The genetics of phenotypic plasticity. II. Response to selection. *J. Evol. Biol.* **4**, 23–50.

30. Lynch M, Lande R. 1993 Evolution and extinction in response to environmental change. In *Biotic Interactions and Global Change*, pp. 234–250.
